# TRIM21–SERPINB5 aids GMPS repression to protect nasopharyngeal carcinoma cells from radiation-induced apoptosis

**DOI:** 10.1186/s12929-020-0625-7

**Published:** 2020-01-31

**Authors:** Panpan Zhang, Xiaomin Li, Qiuping He, Lulu Zhang, Keqing Song, Xiaojing Yang, Qingmei He, Yaqin Wang, Xiaohong Hong, Jun Ma, Na Liu

**Affiliations:** 1 State Key Laboratory of Oncology in South China, Collaborative Innovation Center of Cancer Medicine, Guangdong Key Laboratory of Nasopharyngeal Carcinoma Diagnosis and Therapy, Sun Yat-sen University Cancer Center, 651 Dongfeng Road East, Guangzhou, Guangdong 510060 People’s Republic of China; 2 Max-Planck Center for Tissue Stem cell Research and Regenerative Medicine, Guangzhou Regenerative Medicine and Health Guangdong Laboratory, Guangzhou, 510530 People’s Republic of China; 3 Department of Molecular Diagnostics, Sun Yat-sen University Cancer Center, Guangzhou, China; 4 FireGen Biomedicals Co., LTD, Jiangsu, 215300 China

**Keywords:** Apoptosis, GMPS, Nasopharyngeal carcinoma, SERPINB5, TRIM21, TP53

## Abstract

**Background:**

The main strategy against nasopharyngeal carcinoma (NPC) is radiotherapy. However, radioresistance mediated recurrence is a leading clinical bottleneck in NPC. Revealing the mechanism of NPC radioresistance will help improve the therapeutic effect.

**Methods:**

In this study, the role of TRIM21 (tripartite motif–containing 21) in NPC receiving ionizing radiation was firstly examined both in vivo and in vitro. Mass spectrometry analysis was performed to identify the downstream targets of TRIM21. NPC cells with TRIM21 or SERPINB5 (serpin family B member 5) overexpression or knockout were used to determine the epistatic relationship among SERPINB5, GMPS (guanine monophosphate synthase) and TRIM21. Flow cytometry, co-immunoprecipitation, western blot and immunofluorescence were employed to strengthen the results. Finally, immunohistochemistry using 4 radiosensitive and 8 radioresistent NPC patient samples was perform to examine the association between SERPINB5 or GMPS expression and patient radio-sensitivity.

**Results:**

As an E3 ligase, TRIM21 was highly expressed in NPC. After ionizing radiation, TRIM21 repressed TP53 expression by mediating GMPS ubiquitination and degradation. Overexpression of TRIM21 protected NPC cells from radiation mediated cell apoptosis in vitro and in vivo. Further analysis revealed that TRIM21 mediated GMPS repression was dependent on SERPINB5, and SERPINB5 served as an adaptor which prevented GMPS from entering into the nucleus and introduced TRIM21 for GMPS ubiquitination. Moreover, the in vitro and in vivo results validated the finding that SERPINB5 promoted NPC cell radioresistance, and the radioresistant patients had higher SERPINB5 expression.

**Conclusions:**

Overall, our data showed that TRIM21–SERPINB5-mediated GMPS degradation facilitated TP53 repression, which promoted the radioresistance of NPC cells. This novel working model related to TP53 suppression provided new insight into NPC radioresistence clinically.

## Background

Nasopharyngeal carcinoma (NPC) is a malignant head and neck cancer with apparent regional aggregation [1–3]. Radiotherapy is the most effective treatment strategy against NPC. With modern intensity-modulated radiation therapy, the 5-year overall survival rate of patients with NPC is increased to nearly 80% [4]. However, about 20% of patients with locoregionally advanced disease will have local or regional recurrence, and 90% of the recurrence is in the radiation field [5], owing to radioresistance of the tumor cells. Therefore, revealing the underlying mechanism governing NPC radioresistance would shed light on new clinical therapy and help improve the curative effect.

Upon DNA damage resulting from ionizing radiation or cytotoxic drugs, TP53 will activate the DNA repair system to maintain the integrity of the whole genome, while the apoptotic process will be started if the DNA damage proves irreparable. TP53-governed apoptosis is considered the main cause of ionizing radiation–induced cell death, despite the fact that some cancer cells undergo TP53-independent apoptosis [6, 7]. Therefore, the radioresistant tumor cells are often accompanied by *TP53* mutation or repression, high levels of B-Cell CLL/Lymphoma 2 (*BCL2*), or inhibition of the other apoptosis-related genes [8–12]. In NPC, it has been suggested that latent membrane protein 1 (LMP1), encoded by Epstein–Barr virus, blocks apoptosis and thereby facilitates radioresistance of the tumor cells [13]. Moreover, microRNA-205 inhibits apoptosis by repressing phosphatase and tensin homolog (*PTEN*) expression in NPC [14]. However, the mechanism of NPC radioresistance remains largely unknown.

*TP53* is not frequently mutated in NPC [15, 16]. Consequently, it appears that TP53 expression and its related signaling might be suppressed in radioresistant NPC cells. The protein stability of TP53 is mainly modulated by the interplay between the ubiquitination ligase MDM2 (MDM2 proto-oncogene) and the deubiquitylating enzymes [17, 18]. In normal conditions, TP53 ubiquitination and degradation sustains its low levels in the nucleus. Upon radiation or other genotoxic triggers, TP53 deubiquitylation is accelerated and the TP53 expression level increases correspondingly. Several ubiquitin-specific protease (USP) family members, including USP7 [19], USP10 [20], and USP42 [21], are involved in maintaining TP53 protein stability. However, how TP53 is manipulated in radioresistant NPC cells remains obscure.

Previously, our work indicated that tripartite motif–containing 21 (*TRIM21*) functions as an oncogene during NPC progression [22]. Moreover, TRIM21 can repress TP53 expression by promoting guanine monophosphate synthase (GMPS) ubiquitination and degradation in genotoxic stress conditions [23]. GMPS also interacts with USP7 to mediate gene transcription or H2B deubiquitylation in human cells [18, 24, 25]. Whether the TRIM21–GMPS cascade is conserved in NPC and how this cascade regulates TP53 are all unclear.

Serpin family B member 5 (*SERPINB5*), also known as *MASPIN*, was first reported to function as a tumor repressor gene in breast cancer [26]. However, immunohistochemistry staining in a subsequent study revealed higher SERPINB5 expression levels in patients with breast cancer who had worse prognosis [27], complicating matters. A recent finding suggested that SERPINB5 function is determined by its cellular localization and that SERPINB5 plays a tumor suppressor role only when localized in the nucleus [28]. In NPC, the functional mechanism of SERPINB5 is unknown.

Here, we show that TRIM21 prevented apoptosis in NPC cells after ionizing radiation by mediating GMPS ubiquitination and degradation. Mass spectrometry (MS) and co-immunoprecipitation revealed that SERPINB5 interacts with TRIM21 to facilitate GMPS repression. Moreover, SERPINB5 acts as an adaptor to recruit GMPS protein independent of TRIM21 expression, which was strengthened after radiation. NPC specimens from radioresistant patients had higher SERPINB5 expression levels than radiosensitive patients, suggesting the potential application of SERPINB5 in distinguishing radioresistant patients clinically.

## Materials and methods

### Patients and tumor tissue samples

Tumor samples were obtained from patients with pathologically confirmed NPC (*n* = 12) at Sun Yat-sen University Cancer Center. Radioresistant patients were defined as those with local recurrent disease at the nasopharynx and/or neck lymph nodes at ≤12 months after completion of radiotherapy. Radiosensitive patients were defined as those without local residual lesions at > 3 months and without local recurrent disease > 12 months after completion of radiotherapy.

### Cell lines

NP69, a human immortalized nasopharyngeal epithelial (NPEC) cell line, was cultured in keratinocyte serum-free medium (Invitrogen, Life Technologies, Grand Island, NY, USA) with bovine pituitary extract (BD Biosciences, San Jose, CA, USA). The NPC cell lines 5-8F, 6-10B, C666–1, CNE1, CNE2, HNE1, HONE1, S18, S26, and SUNE1 were cultured in RPMI 1640 medium (Invitrogen) supplemented with 5% fetal bovine serum (FBS, Gibco, Carlsbad, CA, USA). The cells were seeded in 6-well plates the day before transfection, which was performed using Lipofectamine 3000 (Invitrogen), and the cells were harvested 2 days later. For X-ray irradiation, the adhered cells received a 6-Gy dose by an X-ray irradiation apparatus (RS2000, Rad Source, Buford, GA, USA), and were harvested 24 h later.

### RNA extraction and reverse transcription–PCR (RT-PCR)

Total RNA was extracted from the cell lines using TRIzol (Invitrogen). Complementary DNA (cDNA) was synthesized using M-MLV (Moloney murine leukemia virus) reverse transcriptase (Promega, Madison, WI, USA), and amplified using SYBR Green qRT-PCR SuperMix-UDG reagents (Invitrogen) and a CFX96 instrument (Bio-Rad, Hercules, CA, USA). The genes were amplified using the following forward and reverse primers: *GAPDH* (glyceraldehyde-3-phosphate dehydrogenase), 5′-GAAGGTGAAGGTCGGAGT-3′ and 5′-GAAGATGGTGATGGGATTTC-3′; *TRIM21*, 5′-CCCCTCTAACCCTCTGTC-3′ and 5′-GCTAAAGCTCGCTTGCTG-3′; *SERPINB5*, 5′-CATAGAGGTGCCAGGAGC-3′ and 5′-GAACAGAATTTGCCAAAGAA-3′.

### Western blot, co-immunoprecipitation, and immunofluorescence

Total protein was extracted using radioimmunoprecipitation assay lysis buffer (Beyotime, Shanghai, China). Proteins were separated by sodium dodecyl sulfate–polyacrylamide gel electrophoresis and transferred onto polyvinylidene difluoride membranes (Millipore, Billerica, MA, USA). The membranes were then incubated with primary antibodies at 4 °C overnight. After incubation with species-matched secondary antibodies, immunoreactive proteins were detected using chemiluminescence in a gel imaging system (ChemiDoc MP Imaging System, Bio-Rad). The antibodies used were against the following: HA (1:2000, H6908, Sigma-Aldrich, Munich, Germany), FLAG (1:2000, F2555, Sigma-Aldrich), MYC (1:2000, 60,003–2-Ig, Proteintech, Chicago, IL, USA), TRIM21 (1:1000, 12,108–1-AP, Proteintech), SERPINB5 (1:1000, ab182785, Abcam, Cambridge, MA, USA), GMPS (1:1000, 16,376–1-AP, Proteintech), GAPDH (1:2000, ab8245, Abcam), TP53 (1:1000, ab26, Abcam), caspase-3 (1:2000, ab32351, Abcam), lamin B1 (1:1000, ab16048, Abcam), and USP7 (1:5000, 66,514–1-Ig, Proteintech).

For co-immunoprecipitation, cells with ectopic expression of SERPINB5, TRIM21, or GMPS were cultured with MG132 (10 μM, S2619, Selleck Chemicals, Houston, TX, USA) to inhibit proteasome-mediated protein degradation. After 24 h, the cells were harvested for protein purification. The protein was incubated with the corresponding tag antibodies at 4 °C overnight, followed by 3–4-h incubation at 4 °C with protein A/G agarose (20,421, Invitrogen). The beads were then collected for western blot detection. The antibodies used in the co-immunoprecipitation were against the following: HA (1:100, H6908, Sigma-Aldrich), MYC (1:100, 60,003–2-Ig, Proteintech), FLAG (1:100, F2555, Sigma-Aldrich), and immunoglobin G (IgG, 1:100, sc-398,703, Santa Cruz, Dallas, TX, USA).

For immunofluorescence, 1 × 10^5^ cells overexpressing SERPINB5, GMPS, or TRIM21 were seeded and cultured on cover glass, and fixed with methanol after 24 h. The cells were then incubated with tag antibodies at 4 °C overnight, followed by reaction with the corresponding secondary fluorescent antibody (1:500, A-21206, A-21203, Invitrogen) and Hoechst staining (1:5000, H3570, Invitrogen). Images were captured using confocal microscopy (Olympus, Tokyo, Japan).

### Mass spectrometry

HONE1 cells with TRIM21 ectopic expression were harvested for immunoprecipitation. Then, the purified protein underwent MS analysis by Huijun Biotechnology (Guangzhou, China). The enriched protein was obtained by comparing with the IgG group.

### Flow cytometry

For apoptosis analysis, 1 × 10^5^ cells were seeded in 6-well plates. Before the cell density reached about 90%, the cells were collected and stained with annexin V and propidium iodide (PI, C1062, Beyotime), and analyzed by flow cytometry (CytoFLEX 1, Beckman Coulter, Brea, CA, USA). For GFP percentage analysis, 5 × 10^4^ cells with SERPINB5-V2A-GFP overexpression were mixed with 5 × 10^4^ vector control cells and seeded in 6-well plates. Before the cell density reached about 70%, the cells were treated with or without X-ray irradiation, and were harvested 48 h later for GFP analysis.

### Stable cell line establishment and CRISPR gene knockout

The *TRIM21*, *SERPINB5*, and *GMPS* coding sequences were cloned separately into pSin-EF2-puro vector. Stable overexpression cell lines were obtained by puromycin screening and confirmed by western blotting. For CRISPR-mediated gene knockout, the genomic RNAs (gRNAs) were searched (https://zlab.bio/guide-design-resources) and cloned into lentiCRISPRv2 vector. The constructs were transfected into NPC cells, followed by puromycin screening. The surviving cells were confirmed by western blotting. For the single-clone surviving cells, the gDNA was extracted for mutation site identification. The gRNA sequences are as follows. *TRIM21*, 5′-AGCACGCTTGACAATGATGT-3′, *SERPINB5*, 5′-AGCCGAATTTGCTAGTTGCA-3′, and the *SERPINB5* forward and reverse verification primer sequences were 5′-ACTGGGCTCCCGACAATG-3′ and 5′-GCAGGCTGAGGCACAACA-3′, respectively.

### Cell proliferation, colony formation, and cell invasion assays

CCK-8 was used to detect cell proliferative ability. Cells (1 × 10^3^) were seeded in 96-well plates, incubated for 0–4 days, and stained using CCK-8 (Dojindo, Tokyo, Japan). The absorbance was determined at 450 nm using a spectrophotometer.

For the colony formation assay, about 300 cells were seeded in 6-well plates. After 7–10-day culture, the cells were fixed in methanol and stained with crystal violet.

For the cell invasion assay, 3 × 10^3^ cells were seeded in 24-well Transwell chambers (Corning, NY, USA). The medium was supplemented with 10% FBS and placed in the bottom chambers. After 14–18-h culture, the chambers were collected and the cells on lower surface of the chambers were fixed in methanol and stained with crystal violet for observation.

### Clonogenic survival assay

The clonogenic survival assay was performed as previously reported [29]. HONE1 or 5-8F cells were harvested after receiving X-ray radiation, and were re-seeded in 6-well plates and incubated for 12–14 days. Then, the cell colonies were stained with crystal violet and counted. The survival rate of each group was calculated according to the corresponding plating efficiency.

### Animal experiments

B-NDG mice (non-obese diabetes, severe combined immunodeficiency with double knockout of the interleukin-2 receptor gamma chain and protein kinase DNA-activated catalytic genes: NOD-*Prkdc*^*scid*^ *IL2rg*^*tm1*^/Bcgen) of 5–6 weeks old were purchased from Biocytogen Jiangsu Co., Ltd. (Jiangsu, China). The cell groups were all transfected with CMV-luciferase plasmid, and about 1 × 10^6^ cells were injected subcutaneously into the dorsal or ventral flank. The mice were monitored after 7–10 days. Luciferin was diluted to 15 mg/ml using phosphate-buffered saline, and 100 μl of the solution was injected intraperitoneally into each mouse. After 5 min, the mice were anesthetized and observed using an animal imaging system (IVIS Lumina LT, PerkinElmer, Waltham, MA, USA). All animal research was performed in accordance with the detailed rules approved by the Sun Yat-sen University Cancer Center Animal Care and Use Ethics Committee; all efforts were made to minimize animal suffering.

### Immunohistochemistry

Paraffin-embedded patient samples were sectioned and mounted on slides. The slides were incubated at 4 °C overnight with antibody against SERPINB5 (1:200, ab182785, Abcam) or GMPS (1:100, 16,376–1-AP, Proteintech). Then, the sections were incubated with biotinylated secondary antibody bound to a horseradish peroxidase complex. The antibody was visualized by adding 3,3-diaminobenzidine, and the sections were counterstained with hematoxylin.

### Statistical analysis

Statistical analyses were performed using SPSS 17.0 (SPSS Inc., Chicago, IL, USA). All of the data shown are representative of at least three independent experiments, and values are reported as the mean ± SD. Differences between two groups were analyzed using the two-tailed unpaired Student’s *t*-test; *P* < 0.05 was considered significant. The key raw data of the work was uploaded onto the Research Data Deposit public platform with the approval RDD number as RDDB2020000789.

## Results

### TRIM21 served as an oncogene in NPC

**Fig. 1:**
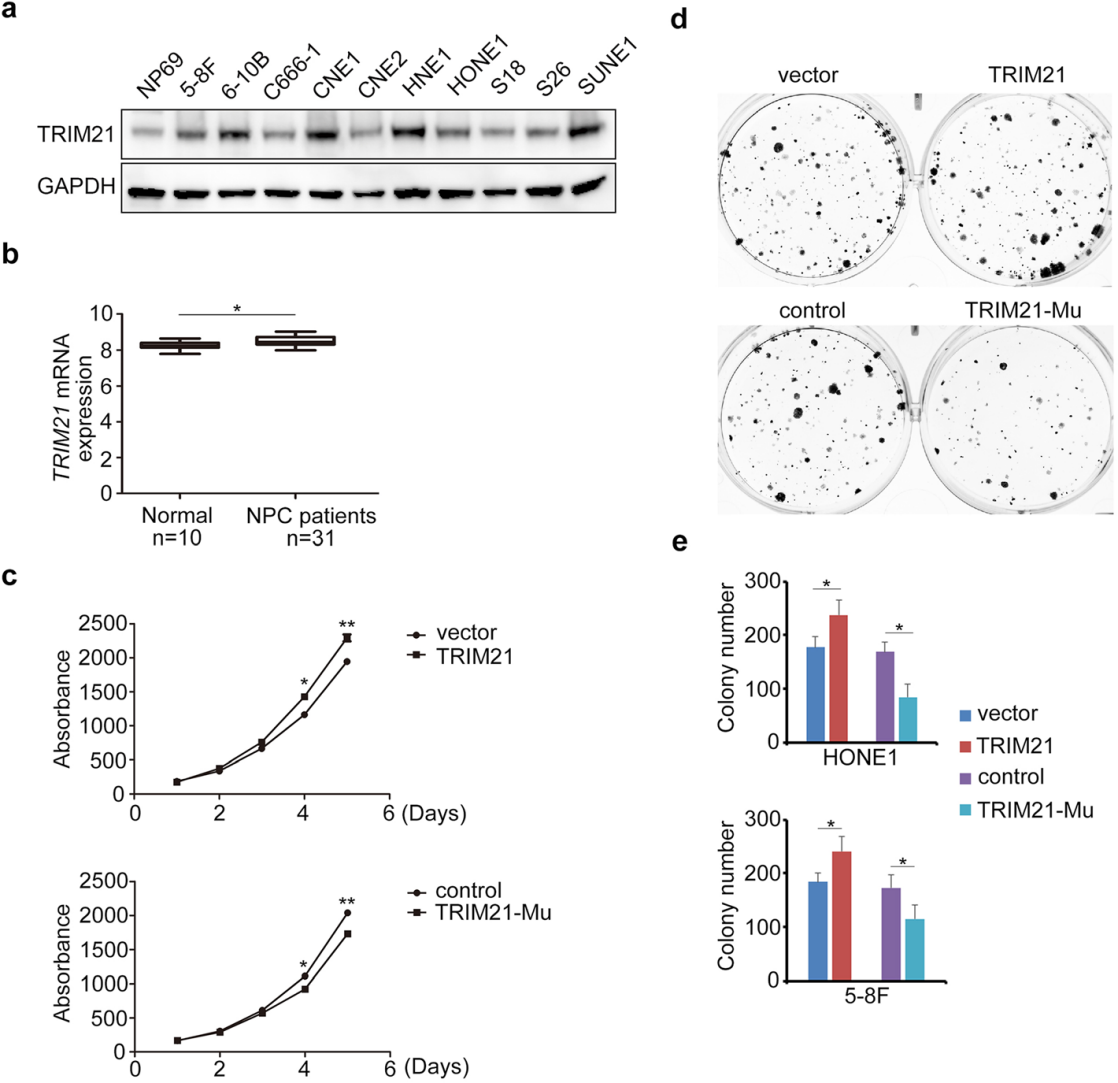
TRIM21 serves as an oncogene in NPC. **a** Western blot detection of TRIM21 expression in normal NP69 cell line and in NPC cell lines. **b** *TRIM21* expression in healthy controls and patients with NPC in the Gene Expression Omnibus (GEO) dataset (81672252). **c** CCK-8 assay of HONE1 cells with *TRIM21* GOF or LOF. **d, e** Colony formation assay and the quantification analysis of NPC cells with *TRIM21* GOF or LOF. *P < 0.05, **P < 0.01, ***P < 0.001. Mu, mutant; ns, not significant

First, we examined the function of TRIM21 in NPC. TRIM21 protein expression levels in NPC cell lines were upregulated (Fig. 1a), as was that in NPC biopsy samples (Gene Series Expression [GSE]81,672,252, Fig. 1b). To explore the function of TRIM21 in NPC, we generated a stable *TRIM21* gain-of-function (GOF) NPC cell line, and *TRIM21* CRISPR (clustered regularly interspaced short palindromic repeats) knockout mutant (loss of function, LOF) NPC cells (Additional file 1: Figure S1a). TRIM21 promoted NPC cell proliferation, which was demonstrated by Cell Counting Kit-8 (CCK-8) and the colony formation assay (Fig. 1c-e). However, there was no sign of TRIM21 involvement in NPC cell invasion (Additional file 1: Figure S1b). Therefore, the data indicate that *TRIM21* functions as an oncogene in NPC.

### TRIM21 repressed GMPS and TP53 expression in NPC

**Fig. 2:**
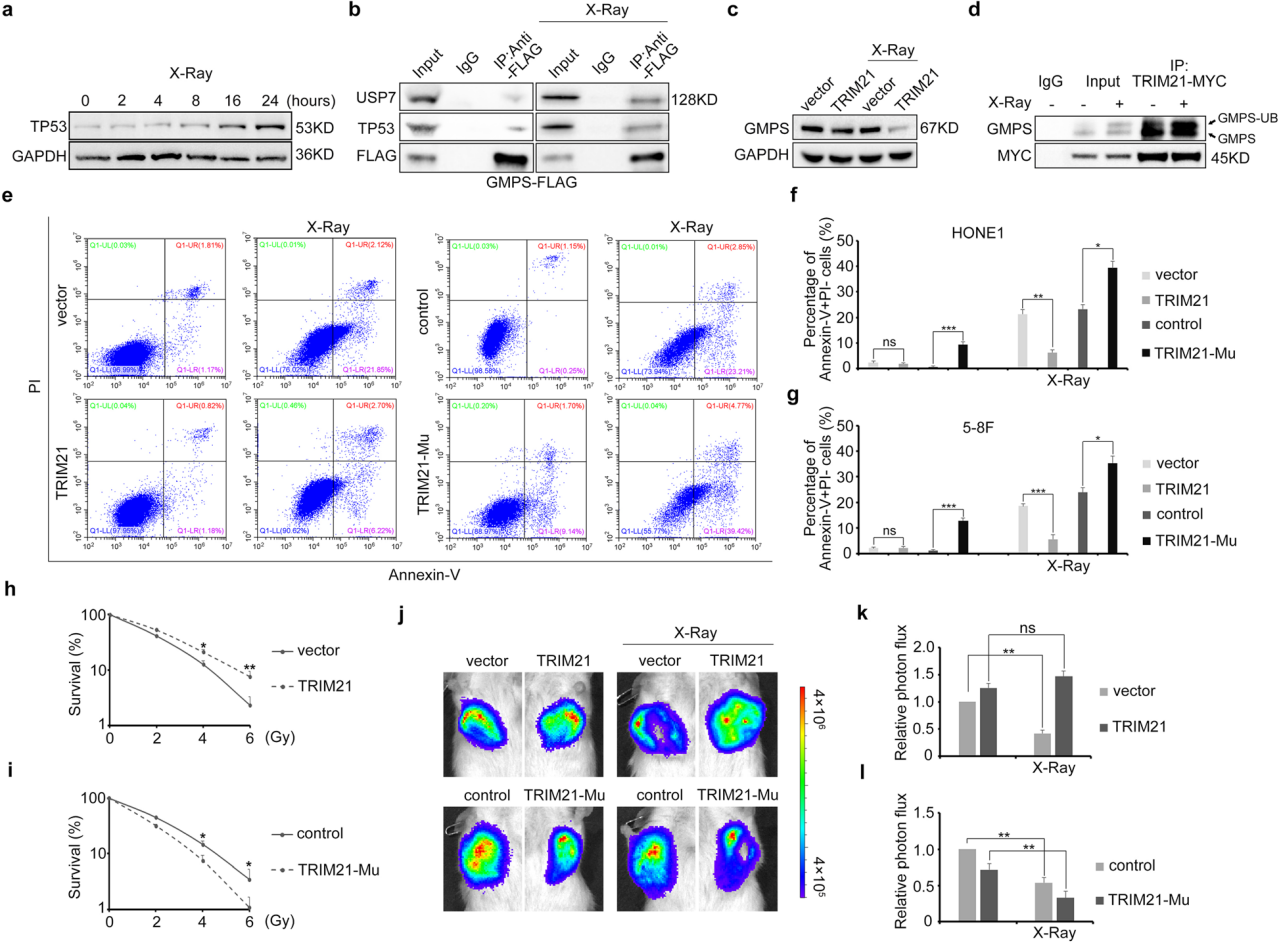
TRIM21 protects NPC cells from radiation-induced apoptosis by manipulating the GMPS–TP53 cascade. **a** Western blot detection of TP53 expression in HONE1 cells after radiation. **b** Co-immunoprecipitation using anti-FLAG antibody following western blot detection of USP7 and TP53 expression in GMPS–FLAG-overexpressing NPC cells with or without X-ray radiation. **c** GMPS expression in TRIM21-overexpressing NPC cells with or without X-ray radiation. **d** Co-immunoprecipitation using anti-MYC antibody following western blot detection of GMPS expression in TRIM21–MYC-overexpressing NPC cells with or without X-ray radiation. **e** Flow cytometry analysis of Annexin V and PI staining in HONE1 cells with *TRIM21* GOF or LOF after X-ray radiation. Annexin^+^PI^−^ cells, which were in the early apoptotic stage, were evaluated and quantified. **f, g** Quantification of the apoptotic HONE1 **f** and 5-8F **g** cells. **h, i** Clonogenic survival assay of HONE1 cells with *TRIM21* GOF **h** or LOF **i**. **j** Absorbance intensity of *TRIM21* GOF and LOF tumor cells and their counterpart controls in mice (*n* = 5 for each group). The tumors were evaluated 2 and 3 weeks, respectively, after implantation, and the mice received radiotherapy (2 Gy daily and a total of 12 Gy) after 2 weeks. **k, l** The absorbance intensity analysis of tumors in mice. For the co-immunoprecipitation assay, the cells were pre-treated with MG132 to avoid GMPS degradation before harvesting. *P < 0.05, **P < 0.01, ***P < 0.001. Mu, mutant; ns, not significant; IP, immunoprecipitation

As TRIM21 protects breast cancer cells from chemotherapy-mediated apoptosis by repressing the GMPS–TP53 cascade, we wondered whether NPC cells share the same mechanism scenario after radiation. X-ray irradiation was followed by an obvious increase in TP53 expression (Fig. 2a). However, this increase was reversed in NPC cells with TRIM21 ectopic expression, and vice versa (Additional file 1: Figure S1c). Then, we examined TP53 mediated immunoprecipitation and found that MDM2 expression decreased after radiation (Additional file 1: Figure S1d). We next expressed FLAG-tagged GMPS in HONE1 cells, followed by anti-FLAG antibody–mediated immunoprecipitation. In the context of X-ray radiation, GMPS bound both USP7 and TP53 (Fig. 2b), and TP53 protein levels were elevated upon GMPS overexpression (Additional file 1: Figure S1e), suggesting that GMPS promotes TP53 protein stability. In addition, TRIM21 downregulated GMPS, especially under the condition of radiation (Fig. 2c).

MS and immunoprecipitation were performed using TRIM21–MYC purified cell lysate. GMPS was included in the MS analysis (Additional file 5: Table S1). Ubiquitinated GMPS was identified in the immunoprecipitated cell lysate with TRIM21–MYC overexpression (Fig. 2d), indicating that radiation facilitated TRIM21-mediated GMPS protein ubiquitination and degradation.

### TRIM21 prevented NPC cell apoptosis in vitro and in vivo

Based on the above findings, we deduced that altered *TRIM21* expression disrupted NPC cell apoptosis. Therefore, HONE1 and 5-8F NPC cells with *TRIM21* GOF or LOF underwent annexin V staining and flow cytometry analysis. As expected, X-ray–irradiated *TRIM21* GOF cells had significantly attenuated early apoptosis, and vice versa (Fig. 2e–g). The clonogenic survival assay showed that TRIM21 elevated the survival rate of NPC cells, while TRIM21 blockage attenuated it (Fig. 2h, i). Moreover, TRIM21 overexpression attenuated active caspase-3 expression (Additional file 1: Figure S1f).

To identify whether manipulating TRIM21 expression would modify NPC cell radiosensitivity in vivo, *TRIM21* GOF or LOF HONE1 cells with luciferase activity were injected subcutaneously into immunodeficient mice. Following X-ray radiation, tumor formation was observed and evaluated. Consistent with the above results, high *TRIM21* expression levels protected the tumor cells from radiation-mediated cell death, whereas *TRIM21* knockout rendered NPC cells radiosensitive (Fig. 2j–l). Therefore, our data demonstrate that TRIM21 plays an essential role in regulating NPC cell radiosensitivity.

### SERPINB5 interacted with TRIM21 to facilitate GMPS repression

**Fig. 3:**
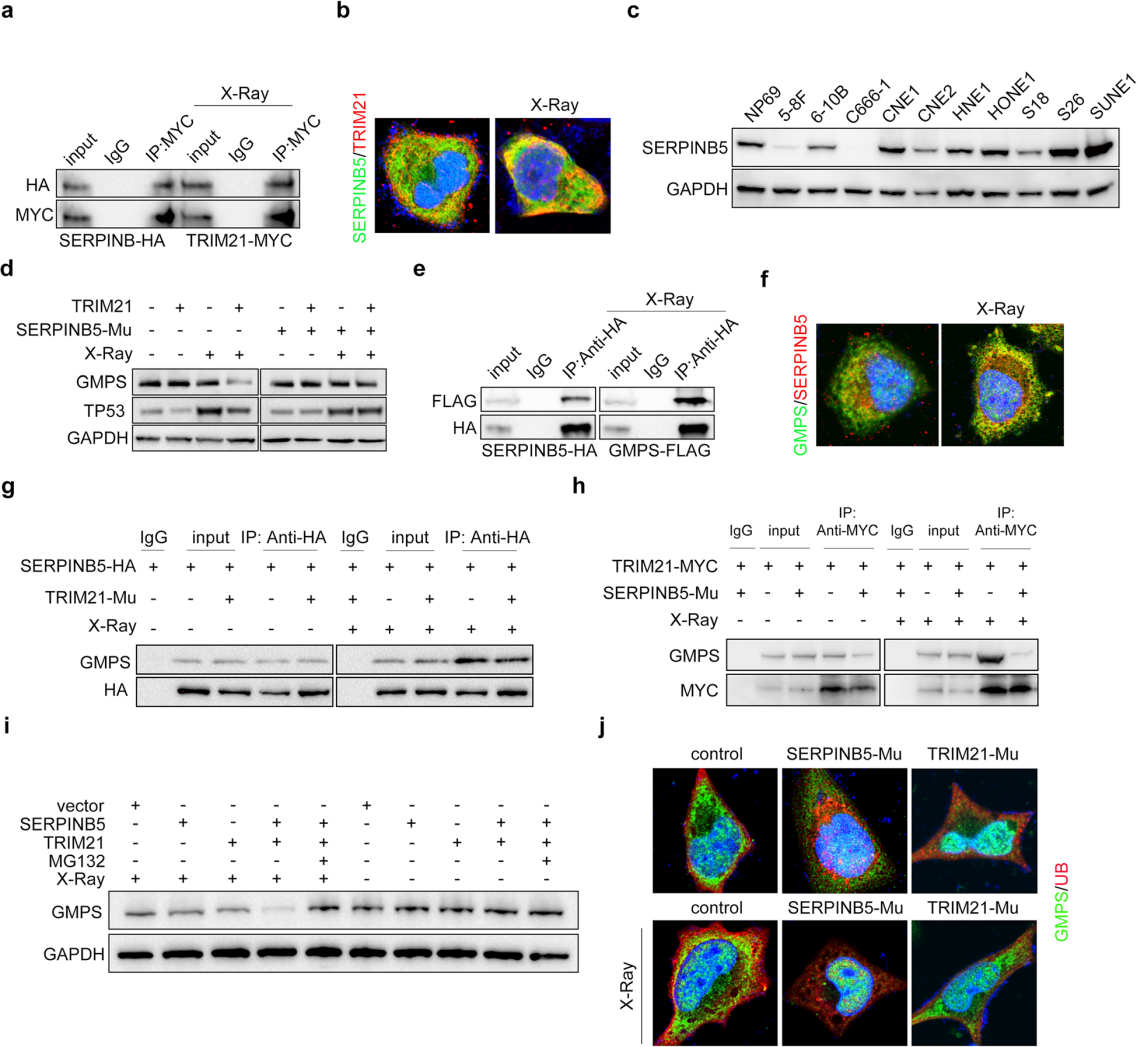
SERPINB5 is indispensable for TRIM21-mediated GMPS–TP53 repression after radiation. **a** Co-immunoprecipitation following western blotting in NPC cells with SERPINB5–HA and TRIM21–MYC overexpression. **b** Immunofluorescence staining analysis of SERPINB5 and TRIM21 in NPC cells. **c** Western blot detection of SERPINB5 expression in normal NP69 cell line and in NPC cell lines. **d** Western blot detection of GMPS and TP53 in NPC cells with TRIM21 GOF or SERPINB5 LOF. **e** Co-immunoprecipitation using anti-HA antibody in NPC cells with SERPINB5–HA and GMPS–FLAG overexpression. **f** Immunofluorescence staining to detect the co-localization of GMPS and SERPINB5 in NPC cells with or without ionizing radiation. **g** GMPS expression in immune-purified protein by anti-HA antibody from NPC cells with or without TRIM21 LOF. **h** GMPS expression in immune-purified protein by anti-MYC antibody from NPC cells with or without SERPINB5 LOF. **i** GMPS expression in NPC cells with TRIM21 or SERPINB5 overexpression. **j** Immunofluorescence staining of overexpressed GMPS and ubiquitin in HONE1 cells with or without ionizing radiation. The localization of GMPS and was evaluated in condition of SERPINB5 or TRIM21 LOF. For the co-immunoprecipitation assay, MG132 was added into the cell medium before cell harvesting to avoid GMPS degradation. *P < 0.05, **P < 0.01, ***P < 0.001

As X-ray radiation accelerated TRIM21-mediated GMPS ubiquitination, we proposed that there are radiation-activated factors that facilitate GMPS degradation. The MS data showed that SERPINB5 was highly enriched (Additional file 5: Table S1). The co-immunoprecipitation showed that TRIM21 interacted with SERPINB5 in NPC cells and that radiation strengthened this interaction (Fig. 3a). Moreover, immunofluorescence staining revealed that SERPINB5 mainly localized in the cytoplasm, along with the colocalized TRIM21 protein (Additional file 2: Figure S2a, Fig. 3b).

To determine the role of SERPINB5 in NPC, we detected its protein level in NPC cell lines first. Surprisingly, *SERPINB5* expression was not consistent in the NPC cell lines (Fig. 3c). Moreover, *SERPINB5* was not significantly different between normal and NPC biopsy samples (Additional file 2: Figure S2b). To explore the function of SERPINB5 in NPC, HONE1 and 5-8F cells, which have higher and lower *SERPINB5* mRNA levels, respectively, were employed. We generated stable *SERPINB5* overexpression cells and CRISPR knockout cells (the transcription start site was mutated [Mu]) (Additional file 2: Figure S2c, S2d). CCK-8 and the colony formation assay revealed that SERPINB5 did not function during NPC cell proliferation (Fig. S2e, S2f). In addition, the Transwell assay showed that SERPINB5 was not related to the cell invasive process (Additional file 2: Figure S2 g).

Then, we asked whether SERPINB5 is involved in TRIM21-mediated GMPS–TP53 repression. Western blotting indicated that TRIM21-mediated TP53 attenuation was dependent on SERPINB5 expression, even in the context of X-ray radiation (Fig. 3d). Next, we examined the counteraction between SERPINB5 and GMPS. GMPS was precipitated by anti-HA (hemagglutinin) antibody, and X-ray radiation promoted the interaction (Fig. 3e). Immunofluorescence indicated that ionizing radiation strengthened the GMPS and SERPINB5 colocalization (Fig. 3f).

Then, we wondered whether the interaction between SERPINB5 and GMPS was dependent on TRIM21. Endogenous GMPS expression was evaluated in cell lysates with *SERPINB5* ectopic expression or *TRIM21* mutation. Our data suggested that SERPINB5 binding of GMPS was dependent on X-ray stimulation, regardless of *TRIM21* expression (Fig. 3g). Correspondingly, the interaction between TRIM21 and GMPS was dependent on SERPINB5 expression, even in the condition of irradiation (Fig. 3h). In addition, GMPS protein was subjected to proteasome-dependent degradation in NPC cells after the radiation, which the concomitant TRIM21 and SERPINB5 overexpression accelerated (Fig. 3i).

### SERPINB5 recruited GMPS and prevented it from entering into the nucleus

According to previous findings, GMPS stabilizes TP53 after entering the nucleus [23]. Therefore, we examined the localization of GMPS in cells with or without ionizing radiation. GMPS localized in both the cytoplasm and the nucleus, while ionizing radiation facilitated GMPS ubiquitination in the cytoplasm, which was not observed in the *TRIM21* mutant cells (Fig. 3j). Moreover, GMPS mainly localized in the nucleus in *SERPINB5* mutant cells after radiation (Fig. 3j). Then, we detected GMPS expression in the cytoplasm and the nucleus. TRIM21-mediated GMPS downregulation in the cytoplasm was dependent on SERPINB5, and GMPS protein tended to localize in the nucleus without SERPINB5 (Additional file 2: Figure S2 h). These data suggest that SERPINB5 is irreplaceable in mediating GMPS ubiquitination by TRIM21.

### SERPINB5 protected NPC cell from apoptosis in vitro and in vivo

**Fig. 4:**
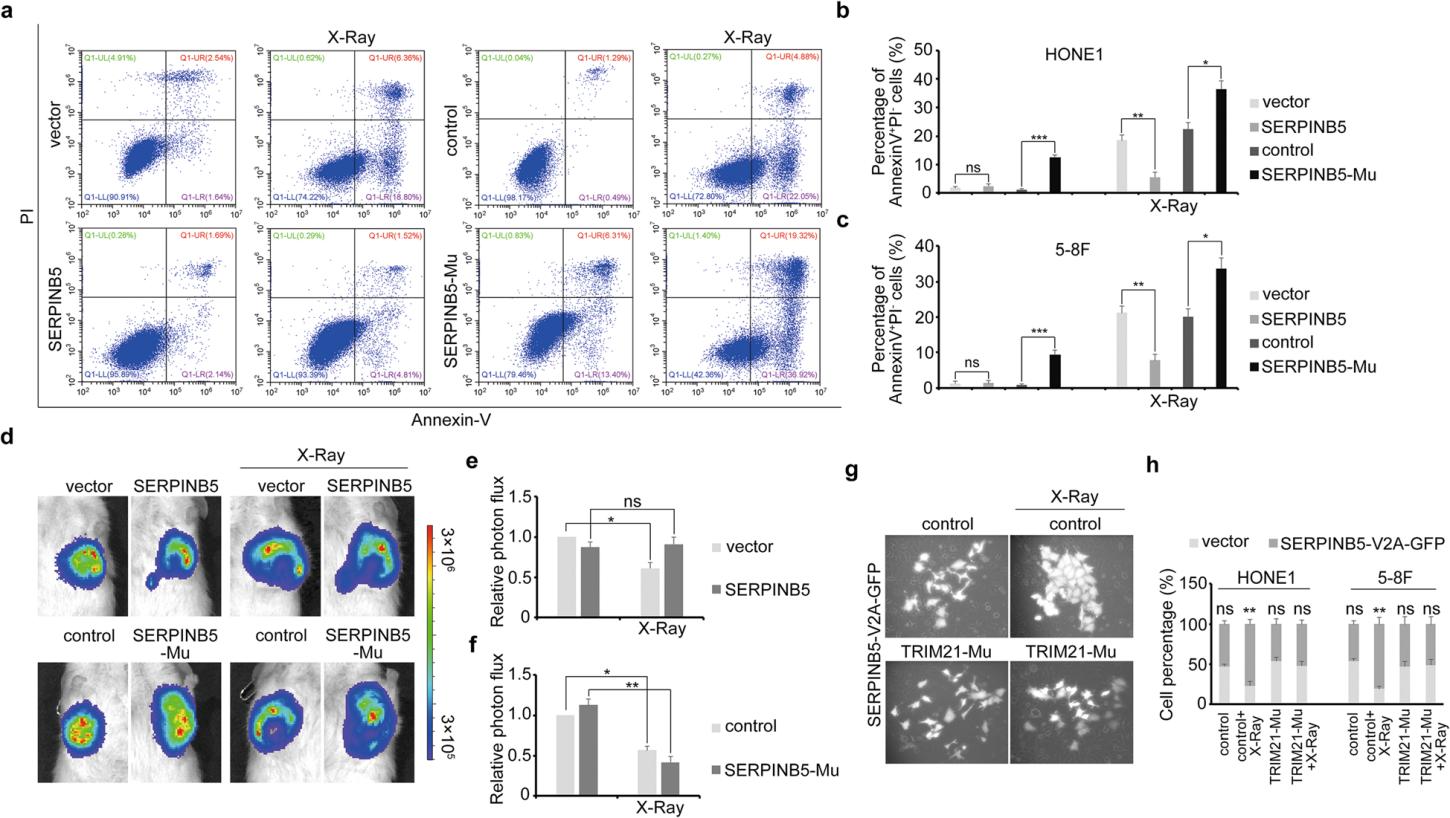
SERPINB5 prevents X-ray radiation–induced NPC cell apoptosis. **a** Flow cytometry analysis of Annexin V and PI staining in HONE1 cells with *TRIM21* GOF or LOF after X-ray radiation. Annexin^+^PI^−^ cells were evaluated and quantified. **b, c** Quantification of apoptotic HONE1 **b** and 5-8F **c** cells. **d** Absorbance intensity of *SERPINB5* GOF (top) and LOF (bottom) tumor cells and their counterpart controls in mice (*n* = 5 for each group). The tumors were evaluated 2 and 3 weeks, respectively, after implantation, and the mice received radiotherapy (2 Gy daily and a total of 12 Gy) after 2 weeks. **e, f** Absorbance intensity analysis of the tumors in mice. **g** GFP expression in HONE1 cells with SERPINB5-V2A-GFP overexpression or TRIM21 knockout. **h** Flow cytometry analysis of GFP^+^ cell percentages in HONE1 and 5-8F cells. *P < 0.05, **P < 0.01, ***P < 0.001

Next, we speculated that SERPINB5 might also play roles in governing NPC cell radiosensitivity. Annexin V staining followed by flow cytometry analysis revealed that ectopic expression of SERPINB5 protected the tumor cells from radiation-induced apoptosis, and vice versa (Fig. 4a–c). The clonogenic survival assay showed that tumor cells lacking *SERPINB5* became sensitive and vulnerable to radiation (Additional file 3: Figure S3a, S3b). In addition, *SERPINB5* mutation completely blocked the radioresistant effect of TRIM21 (Additional file 3: Figures S3c, S3d), suggesting that TRIM21 acts through SERPINB5 to manipulate tumor cell radiosensitivity.

To demonstrate the effect of SERPINB5 in vivo, HONE1 cells with *SERPINB5* GOF or LOF were injected subcutaneously into immunodeficient mice, followed by regular X-ray radiation and observation. SERPINB5 rendered the tumor cells resistant and refractory to radiotherapy (Fig. 4d–f). To confirm the radioresistant role of SERPINB5 in NPC, we constructed a V2A vector system with simultaneous SERPINB5 and GFP (green fluorescent protein) co-expression. The dynamic expression of GFP was evaluated after X-ray radiation in SERPINB5-V2A-GFP expression cells that had been mixed with their control cell counterparts. The percentage of GFP-positive cells increased significantly after radiation, while this increase was abrogated in the cells without *TRIM21* (Fig. 4g, h), indicating that SERPINB5-mediated tumor cell radioresistance is dependent on TRIM21. These data demonstrate that SERPINB5 and TRIM21 function together as pivotal regulators during NPC radiotherapy.

**Fig. 5:**
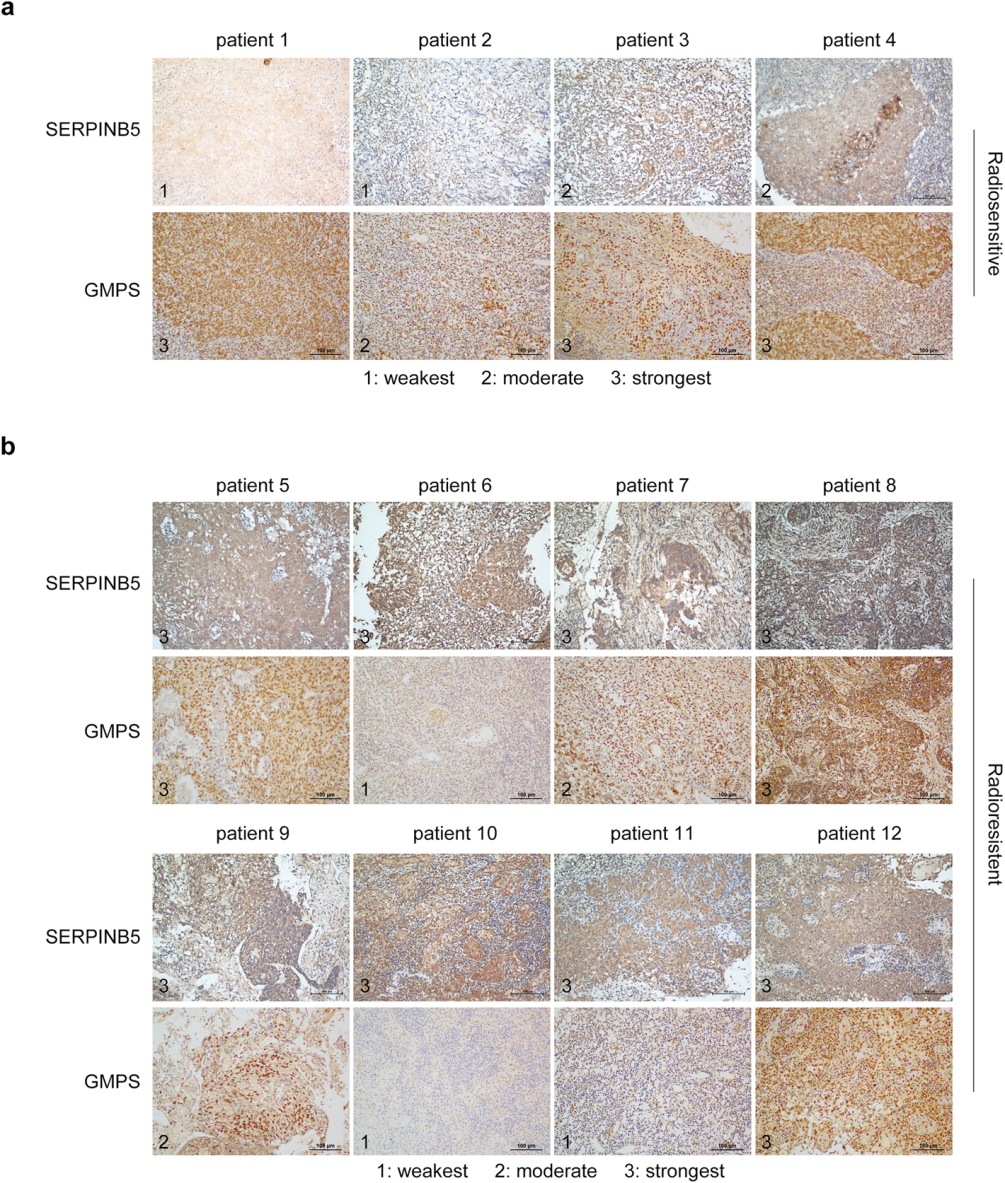
SERPINB5 expression increases in radioresistant NPC patients. **a, b** Immunohistochemistry staining of SERPINB5 and GMPS in radiosensitive **a** and radioresistant **b** patients. Based on the staining intensity, the images are divided into three grades from weakest to strongest (from 1 to 3, respectively)

As shown above, patients with NPC had upregulated *TRIM21* expression, while *SERPINB5* expression varied between patients. As the patients had varied outcomes after radiotherapy, we hypothesized that the SERPINB5 expression level determines the radiosensitivity of patients with NPC. To prove this, we used specimens from four radiosensitive patients and eight patients refractory to radiotherapy. Immunohistochemistry staining revealed that all radioresistant NPC samples had higher SERPINB5 expression levels (Fig. 5a, b). Moreover, GMPS expression correlated negatively with SERPINB5 somewhat, illustrating the validity of the TRIM21–SERPINB5–GMPS signaling axis in NPC.

In general, our work reveals the regulatory mechanism of TRIM21-mediated GMPS–TP53 repression in NPC, emphasizing the critical role of SERPINB5 during this process. SERPINB5 could recruit free GMPS protein in the cytoplasm, presenting it to TRIM21 for ubiquitination and protein degradation, and X-ray radiation accelerated this process. The decreased expression of GMPS subsequently promoted TP53 degradation, which prevented radiotherapy-induced apoptosis (Additional file 4: Figure S4).

## Discussion

Radioresistance is one of the main obstacles in NPC clinical therapies. However, the mechanism of NPC radioresistance has remained obscure to date. In modern therapeutic strategies against NPC, all patients receive definitive radiotherapy of similar intensity [30–33] despite their tumor heterogeneity. Therefore, some patients with high radiosensitivity experience adverse effects from the radiation, while some patients with low radiosensitivity face the risk of recurrence after radiation. Distinguishing the radioresistant patients is one of the main difficulties restricting improvement of the cure rate. Our findings provide a molecular marker for predicting the radiosensitivity of patients with NPC before treatment.

Our data suggest that *SERPINB5* does not influence NPC progression in normal conditions, while serving as an oncogene after radiation. We demonstrate for the first time that SERPINB5, which mainly localizes in the cytoplasm, functions as an adaptor to bind and prevent GMPS protein from entering the nucleus, and prompts GMPS ubiquitination by interacting with TRIM21. The stimulation by X-ray radiation strengthened this process in NPC. These findings stress the pivotal role of SERPINB5 in mediating GMPS–TP53 cascade repression in radioresistant NPC cells. However, how SERPINB5 detects the radiation signal remains unclear.

Our data reveal that TRIM21 promotes NPC progression in normal conditions, suggesting that it is also involved in other signaling axes in NPC. GMPS was originally found to fuel cancer progression by mediating guanine nucleotide synthesis [34, 35]. The Cancer Genome Atlas (TCGA) dataset showed upregulated GMPS expression in various cancers, including NPC. Considering GMPS expression was not decreased or even elevated in some of the radioresistant patients in the present study, we believe that GMPS plays multiple roles in NPC. Therefore, unlike SERPINB5, GMPS is not a suitable marker for identifying radioresistant patients with NPC.

## Conclusion

In summary, our work establishes a novel working model related to TP53 suppression in radioresistant NPC cells, and highlights the important potential application of SERPINB5 in predicting the radiosensitivity of patients with NPC.

## Supplementary information

**Additional file 1: Figure S1.** The TRIM21–GMPS signaling cascade regulated TP53. (a) Western blot detection of TRIM21 expression in *TRIM21* overexpression or knockout HONE1 cells. (b) Transwell assay of HONE1 cells with *TRIM21* GOF or LOF. (c) TP53 expression in NPC cells with *TRIM21* GOF or LOF after X-ray radiation. (d) TP53 mediated immune-precipitation and western blot detection of USP7 and MDM2 expression. (e) TP53 expression in NPC cells with *GMPS* GOF or *TRIM21* LOF after X-ray radiation. (f) Cleaved caspase-3 expression in HONE1 cells with *TRIM21* overexpression

**Additional file 2: Figure S2.** SERPINB5 does not affect NPC progression in normal conditions. (a) Immunofluorescence staining of SERPINB5–HA in HONE1 cells. (b) *SERPINB5* expression in healthy controls and patients with NPC in the GEO dataset (81672303). (c) CRISPR-mediated *SERPINB5* knockout NPC cells. Bolded, larger typeface indicates the mutated sequences. (d) SERPINB5 expression in *SERPINB5* overexpression or knockout HONE1 cells. (e) CCK-8 assay of NPC cells with *SERPINB5* GOF (top) or LOF (bottom). (f) Colony formation assay of NPC cells with SERPINB5 GOF (top) or LOF (bottom). (g) Transwell assay of NPC cells with *SERPINB5* GOF (top) or LOF (bottom). (h) GMPS expression in cytoplasm (left) or nucleus (right) of HONE1 cells with *TRIM21* overexpression or *SERPINB5* LOF. Wt, wild-type; Mu, mutant. ns, not significant

**Additional file 3: Figure S3.** SERPINB5 is essential for TRIM21-mediated NPC cell survival after radiation. (a, b) The survival rates of HONE1 cells with *SERPINB5* GOF (a) or LOF (b) after radiation. (c, d) The survival rates of HONE1 (c) or 5-8F (d) cells with *SERPINB5* knockout and *TRIM21* GOF. Mu, mutant.

**Additional file 4: Figure S4.** The working model of TRIM21–SERPINB5-mediated GMPS–TP53 repression in NPC cells after X-ray radiation. UB, ubiquitin

**Additional file 5.: Table S1.** Mass spectrometry analysis of the lysate from TRIM21-MYC purified cells.

## Data Availability

All of the data generated during this study are included in this article and its supplementary files.

## Abbreviations

CCK-8: Cell Counting Kit-8
CRISPR: Clustered regularly interspaced short palindromic repeats
GMPS: Guanine monophosphate synthase
GOF: Gain-of-function
LMP1: Latent membrane protein 1
LOF: Loss-of-function
MDM2: MDM2 proto-oncogene
NPC: Nasopharyngeal carcinoma
PTEN: Phosphatase and tensin homolog
SERPINB5: Serpin family B member 5
TCGA: The Cancer Genome Atlas
TRIM21: Tripartite motif–containing 21
USP: Ubiquitin-specific protease
