# Supplementary figures and images for: TRIM21–SERPINB5 aids GMPS repression to protect nasopharyngeal carcinoma cells from radiation-induced apoptosis
Source: J Biomed Sci. 2020 Jan 31;27:30. doi: 10.1186/s12929-020-0625-7 (PMC6995195; doi:10.1186/s12929-020-0625-7)

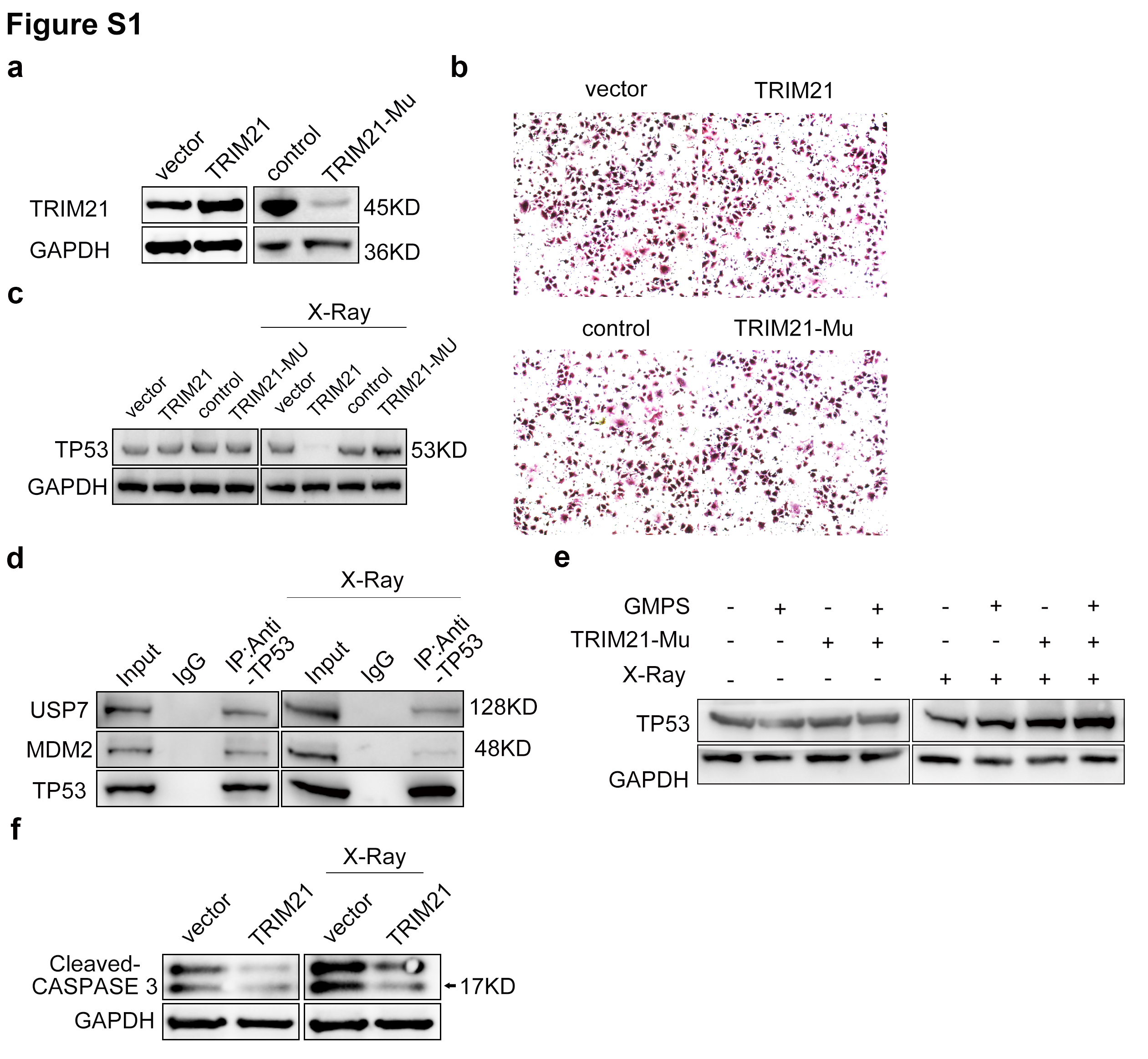

Supplement: Supplementary file 1 — Additional file 1: Figure S1. The TRIM21–GMPS signaling cascade regulated TP53. (a) Western blot detection of TRIM21 expression in TRIM21 overexpression or knockout HONE1 cells. (b) Transwell assay of HONE1 cells with TRIM21 GOF or LOF. (c) TP53 expression in NPC cells with TRIM21 GOF or LOF after X-ray radiation. (d) TP53 mediated immune-precipitation and western blot detection of USP7 and MDM2 expression. (e) TP53 expression in NPC cells with GMPS GOF or TRIM21 LOF after X-ray radiation. (f) Cleaved caspase-3 expression in HONE1 cells with TRIM21 overexpression [file 12929_2020_625_MOESM1_ESM.jpg]

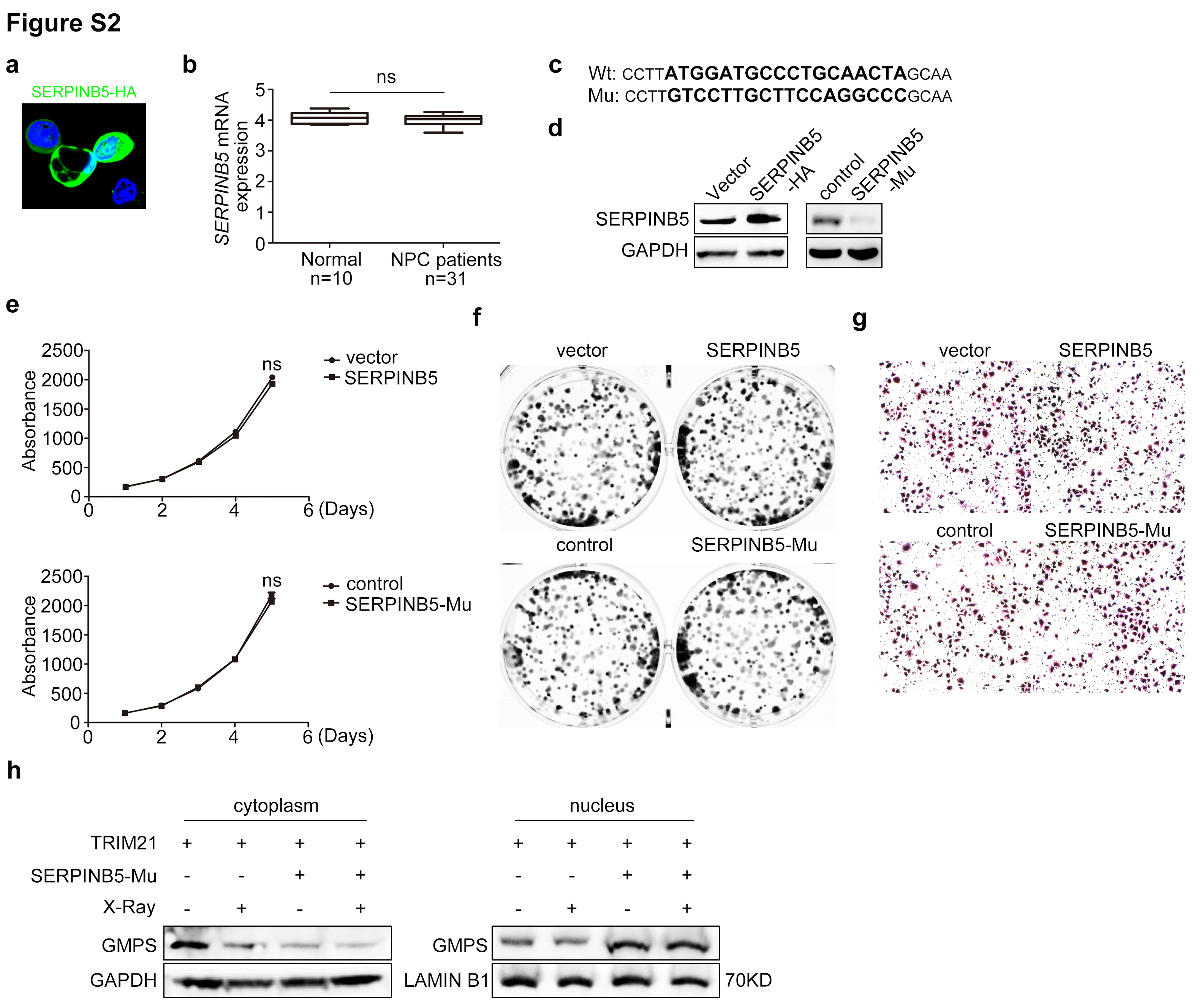

Supplement: Supplementary file 2 — Additional file 2: Figure S2. SERPINB5 does not affect NPC progression in normal conditions. (a) Immunofluorescence staining of SERPINB5–HA in HONE1 cells. (b) SERPINB5 expression in healthy controls and patients with NPC in the GEO dataset (81672303). (c) CRISPR-mediated SERPINB5 knockout NPC cells. Bolded, larger typeface indicates the mutated sequences. (d) SERPINB5 expression in SERPINB5 overexpression or knockout HONE1 cells. (e) CCK-8 assay of NPC cells with SERPINB5 GOF (top) or LOF (bottom). (f) Colony formation assay of NPC cells with SERPINB5 GOF (top) or LOF (bottom). (g) Transwell assay of NPC cells with SERPINB5 GOF (top) or LOF (bottom). (h) GMPS expression in cytoplasm (left) or nucleus (right) of HONE1 cells with TRIM21 overexpression or SERPINB5 LOF. Wt, wild-type; Mu, mutant. ns, not significant [file 12929_2020_625_MOESM2_ESM.jpg]

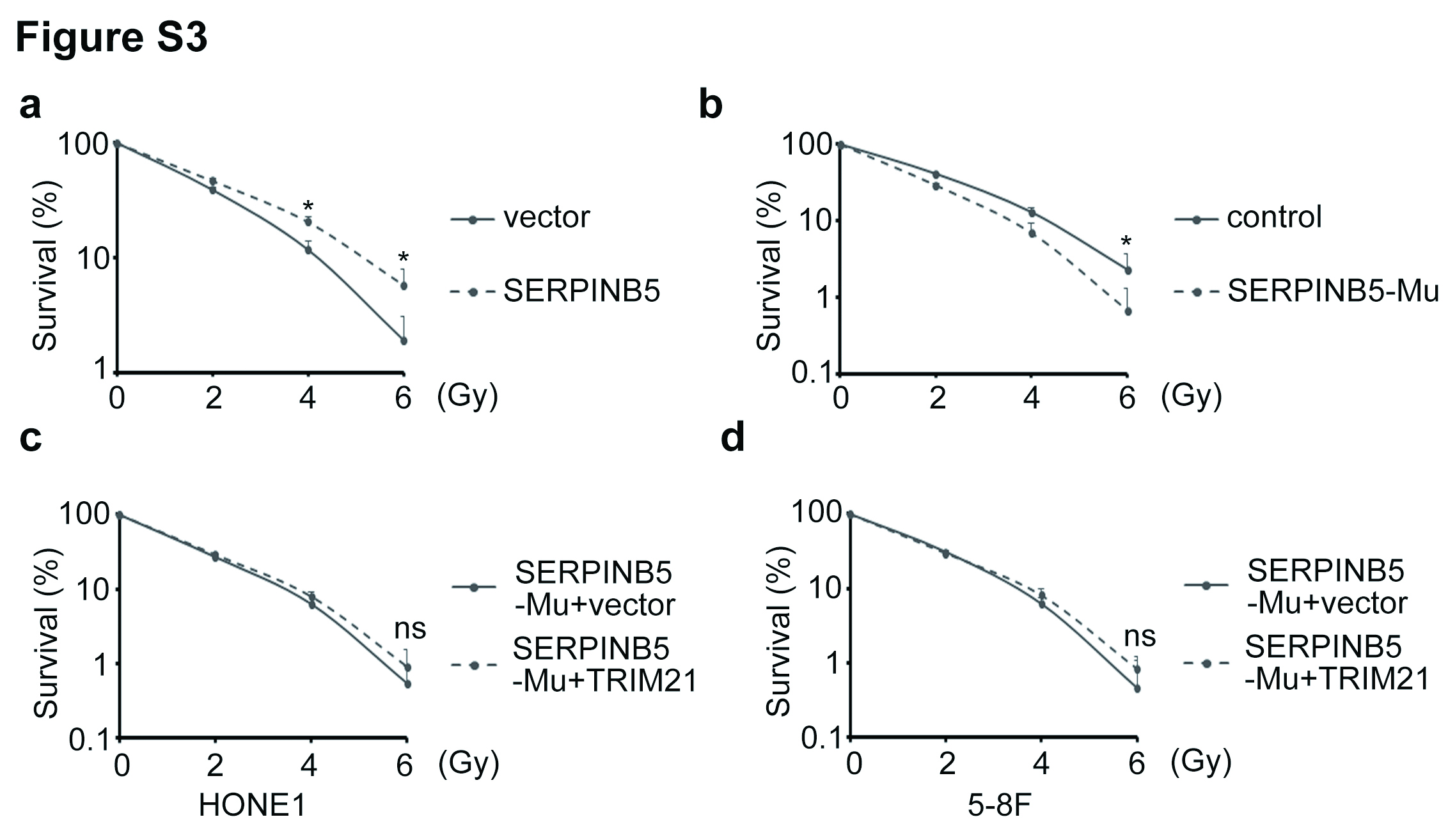

Supplement: Supplementary file 3 — Additional file 3: Figure S3. SERPINB5 is essential for TRIM21-mediated NPC cell survival after radiation. (a, b) The survival rates of HONE1 cells with SERPINB5 GOF (a) or LOF (b) after radiation. (c, d) The survival rates of HONE1 (c) or 5-8F (d) cells with SERPINB5 knockout and TRIM21 GOF. Mu, mutant. [file 12929_2020_625_MOESM3_ESM.jpg]

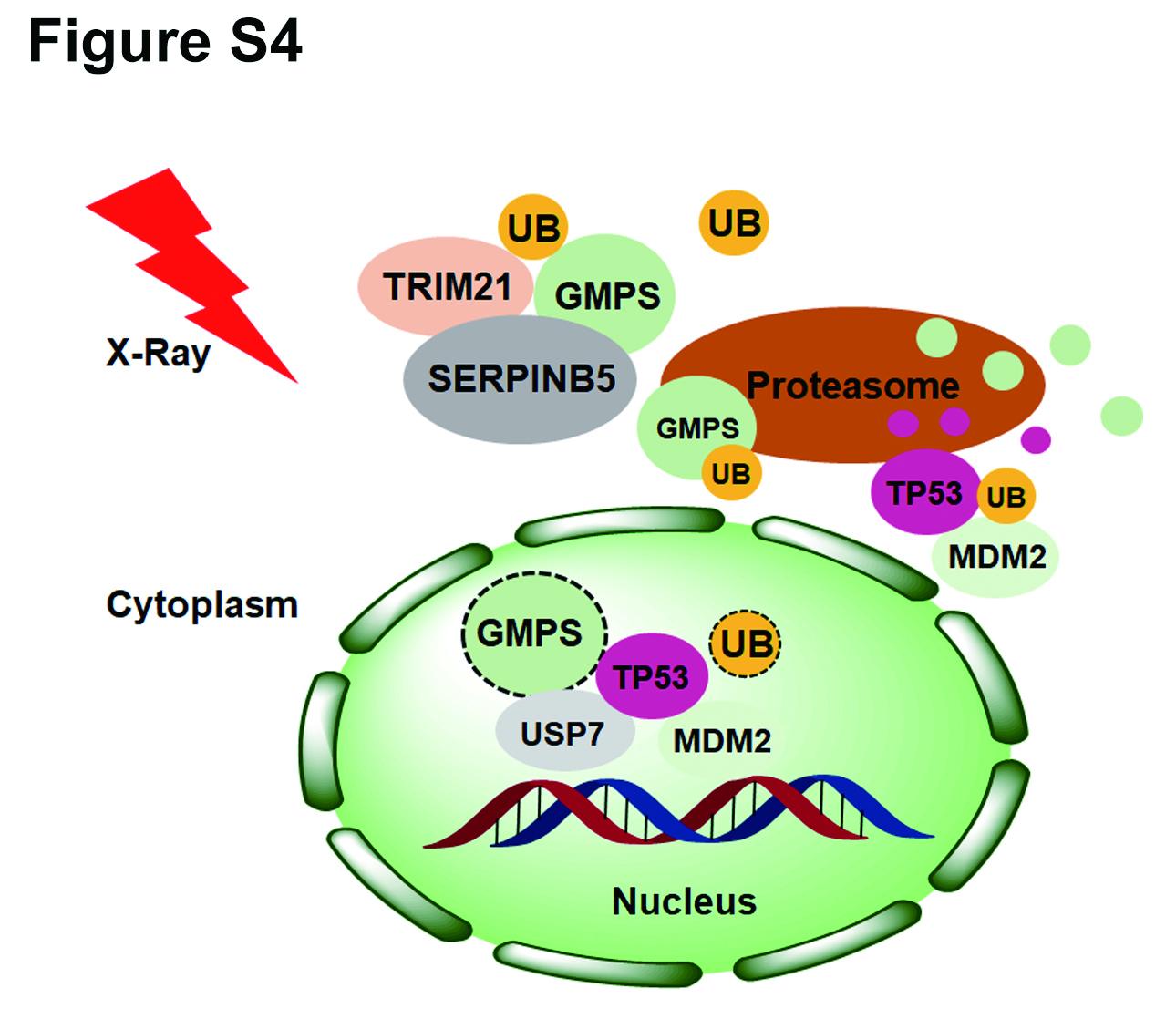

Supplement: Supplementary file 4 — Additional file 4: Figure S4. The working model of TRIM21–SERPINB5-mediated GMPS–TP53 repression in NPC cells after X-ray radiation. UB, ubiquitin [file 12929_2020_625_MOESM4_ESM.jpg]
